# Compact Shielding of Graphene Monolayer Leads to Extraordinary SERS-Active Substrate with Large-Area Uniformity and Long-Term Stability

**DOI:** 10.1038/srep17167

**Published:** 2015-11-30

**Authors:** Xiangjiang Liu, Jiajun Wang, Yichen Wu, Tianren Fan, Yang Xu, Longhua Tang, Yibin Ying

**Affiliations:** 1 College of Biosystems Engineering and Food Science, Zhejiang University, Hangzhou 310058, China; 2 State Key Laboratory of Modern Optical Instrumentation, College of Optical Science and Engineering, Zhejiang University, Hangzhou 310027, China; 3 Institute of Microelectronics and Optolectronics, Zhejiang University, Hangzhou 310027, China

## Abstract

Surface-enhanced Raman scattering (SERS) can significantly boost the inherently weak Raman scattering signal and provide detailed structural information and binding nature of the molecules on the surface. Despite the long history of this technology, SERS has yet to become a sophisticated analytical tool in practical applications. A major obstacle is the absence of high-quality and stable SERS-active substrate. In this work, we report a monolayer graphene-shielded periodic metallic nanostructure as large-area uniform and long-term stable SERS substrate. The monolayer graphene acting as a corrosion barrier, not only greatly enhanced stability, but also endowed many new features to the substrate, such as alleviating the photo-induced damages and improving the detection sensitivity for certain analytes that are weakly adsorbed on the conventional metallic substrates. Besides, our fabrication strategy were also capable of fabricating the reproducible SERS sensing spots array, which may serve as a promising high-throughput or multi-analyte sensing platform. Taken together, the graphene-shielded SERS substrate holds great promise both in fundamental studies of the SERS effect and many practical fields.

Surface-enhanced Raman scattering (SERS) exploites the enhanced localized surface plasmon resonance (LSPR) induced by incident light in metallic nanostructures, which can boost the inherently weak Raman scattering to single-molecule detection level^1^,^2^. Despite its long history^3^, SERS has not yet become a sophisticated tool for practical applications^4^. A major obstacle is lack of a simple approach to fabricate uniform and stable SERS substrates over large area. Historically, roughened electrodes or metallic nanoparticles have been extensively used as SERS substrates, but hardly any reliable quantitative results can be obtained from such random nanostructures, whose properties vary from experiment to experiment. The lithography-based method can fabricate highly ordered SERS substrates^5^,^6^,^7^,^8^, But their high cost in facilities and complexity in preparation steps pose a problem for any practical applications^9^. More cost-effective technique that allows precise control over the surface is still desirable. More importantly, the stability of the SERS substrate is the other concern. The metallic nanostructures are often suffers from oxidation and corrosion, both leading to degradation of the plasmonic characteristics and change of the surface morphology, thereby compromising effective generation of SERS.

Various strategies have been proposed to improve the stability of SERS substrate. By coating the surface with an inert thin layer (SiO_2_^10^,^11^, TiO_2_^12^, Al_2_O_3_^13^, MnO_2_^14^, *etc.*), the oxidation and corrosions of SERS substrate can be prevented. This approach also helps to reduce the fluctuation of SERS signal caused by photocarbonization, photobleaching or metal-catalyzed site reactions, due to the isolation of the substrate and analyte. A more reproducible SERS signal can be expected. However, this approach is an over delicate technique. Given the rapid decay of the LSPR from surface, the inert layer should be thin enough (a few nanometers) to avoid significant sacrifice of the SERS strength. Nevertheless, the coating layer must be extremely uniform, since tiny variation in the thickness can cause a huge fluctuation in the SERS signal^15^. But, the deposition of a uniform ultra-thin layer remains challenging, requiring sophisticated experimental skills, meticulous treatments or long reaction time. Therefore, it is crucial to develop alternative approach to preserve the SERS activity of the substrate.

To overcome these limitations, we designed a graphene-shielded periodic metallic nanostructure as large-area uniform and long-term stable SERS substrate. Graphene is a mechanically strong and chemical inert atomic monolayer. It has uniform thickness and is impenetrable to most gas molecules and liquids^16^,^17^. Thus, a hybrid graphene-covered metallic surface, which was firstly proposed by Song *et al.* as a high-resolution bio/nanosensing platform^18^, is later proved to be also effective in suppressing metal oxidation and corrosion^19^,^20^,^21^,^22^,^23^,^24^. At the moment, the industrial-scale graphene film grown by chemical vapor deposition (CVD) are widely available^25^,^26^,^27^. By passivating the SERS substrate with a monolayer graphene, we found that the substrate exhibited a significantly enhanced physical/chemical stability. Meanwhile, due to the unique structure of graphene, the graphene not only preserved excellent SERS activity of the substrate, but also endowed the conventional SERS substrates with some new features. Furthermore, we also applied this approach to prepare graphene-shield SERS sensing spots array, since sensor array is a verified efficient detection method in high-throughput analysis^28^,^29^,^30^.

## Results

**Figure 1:**
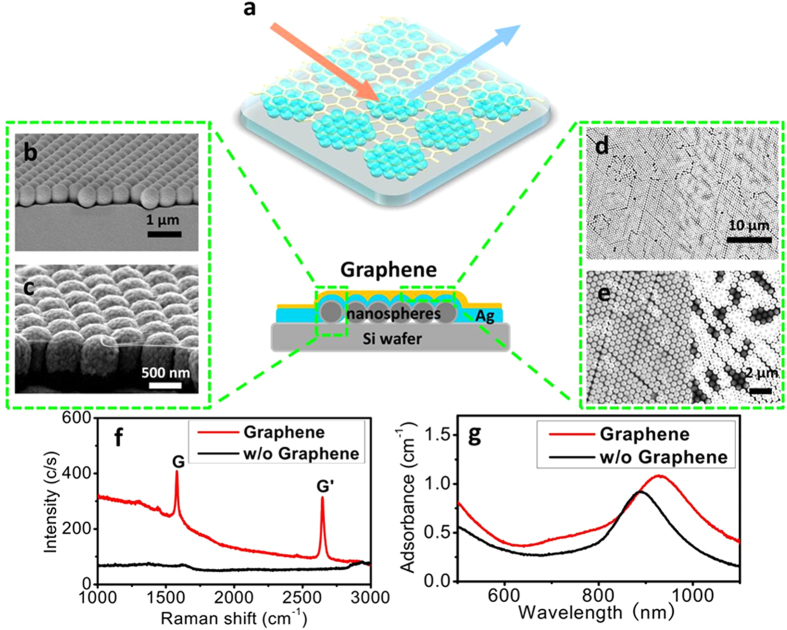
Schematic illustration of the graphene-shielded SERS substrate array (a). The cross-section (**b**,**c**) and the top-view (**d**,**e**) SEM images of the graphene-shielded SERS substrate array; the Raman spectra (**f**) and adsorption spectra (**g**) of the metal-coated nanospheres substrates with or without graphene coating.

Figure 1a schematically depicted the as-prepared graphene-shielded SERS substrate. In this work, a standardized metal-coated nanospheres arrays (MCNAs)^31^ were employed as the SERS substrate. (for detail see Figure S1 in the Supporting Information). Briefly, a monolayer of nanospheres arrays (silica or polystyrene) was firstly assembled at the water-air interface and then transferred onto a clean silica wafer (~1 × 1 cm^2^). Subsequently, a ~200 nm thick silver film was deposited on the nanospheres array, resulting in a MCNAs SERS substrate. Finally, a CVD-grown graphene film was transferred onto the above MCNAs substrate via a wet transfer technique.

Some typical graphene-shielded SERS substrates are shown in Figure S2. Scanning electron microscopy (SEM) was used to characterize the morphology of as-prepared graphene-shielded MCNAs substrates. As shown in Fig. 1b, an ordered metal-coated nanospheres array was observed in the cross section of the prepared substrate, which was arranged into close-packed (111) plane on the Si wafer. After graphene transfer step, a monolayer graphene closely attached on the MCNAs can be easily identified (Fig. 1c). Furthermore, the top-view SEM image was used to verify the morphology of the transferred monolayer graphene. A large-area uniform layer of graphene could be clearly observed in Fig. 1d,e. Besides, as indicated by Fig. 1e, no sign of morphology change was noticed for the graphene-covered area on the MCNAs (additional SEM images shown in Figure S2–S5). But, after the transfer procedures, the uncovered area shows some defects in the nanospheres array, suggesting the enhancement of mechanical stiffness of the substrate by the monolayer graphene.

Raman spectroscopy was used to characterize the graphene-shielded SERS substrate. As plotted in Fig. 1f, the sharp G-band (1570 cm^−1^) and G’-band (2625 cm^−1^) of graphene were clearly observed in the graphene-covered substrate. On the contrary, these two bands were absence for the uncovered MCNAs. Further, the absence of D-band at 1345 cm^−1^ and D’-band at 1625 cm^−1^ (originating from disordered graphene film)^4^, as well as the relatively higher-intensity of G’ band to G-band^32^, indicated the high quality and monolayer feature of the transferred graphene film. All these results indicated the successfully transfer of the monolayer graphene. In addition, Fig. 1g plots the adsorption spectra of the substrate with and without graphene layers. The broadening and red-shifting of the spectra was found for the graphene-covered substrate, as well as increased the absolute adsorption compared with uncovered substrate. This can be attributed to the graphene acting as a lossy dielectric in the visible and near-infrared range and plasmonic nanostructure embedded in a higher refractive index medium results in a longer resonance wavelength^33^.

**Figure 2:**
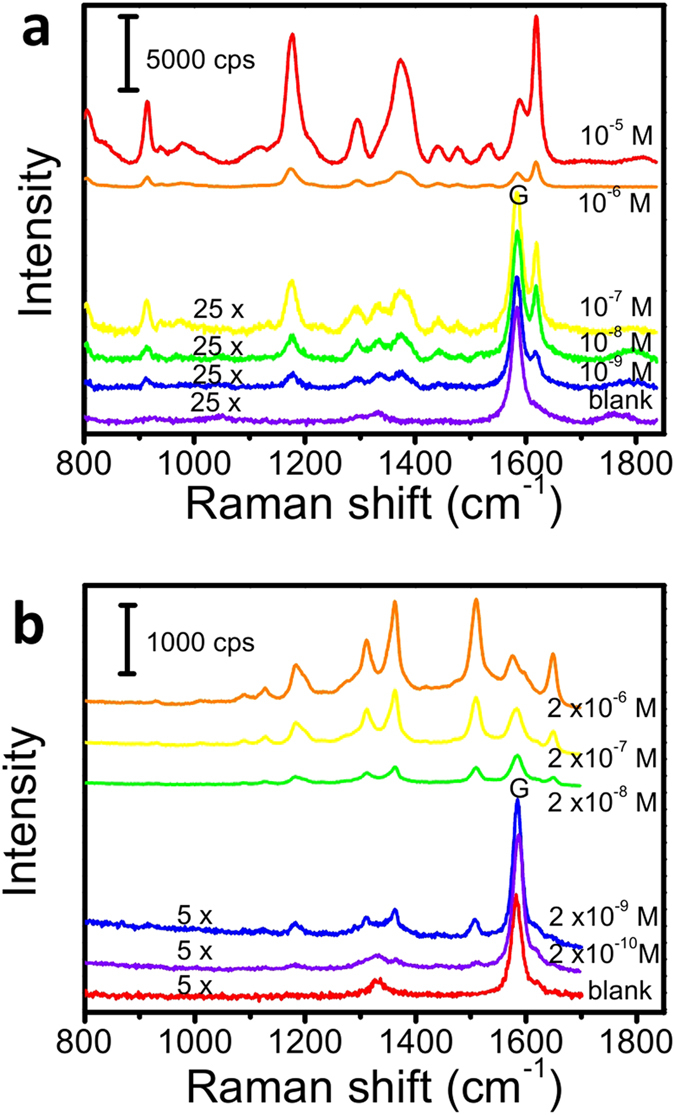
Raman spectra recorded from graphene-shielded substrates immersed in different concentration of CV (a) and R6G (b) solutions (50× objective, average of 16 spectra with 1s acquisition time).

To evaluate the SERS activity of the graphene-shielded substrates, crystal violet (CV) and rhodamine 6G (R6G) were used to probe the substrate. The corresponding spectra are displayed in Fig. 2a,b. A positive correlation between the band intensities and the concentrations of the dyes was observed. We noticed that certain Raman bands were distantly visible when CV and R6G concentrations were as low as 10^−9^ M and 2 × 10^−10^ M, respectively. The enhancement factor (EF) of the substrate was estimated to be ~10^7^, by calculating the ratio of SERS intensity to the corresponding normal Raman intensity. This value is among the highest EFs measured on similar kind of two-dimension immobilized SERS substrate^31^,^34^,^35^. To evaluate the influence of graphene to the SERS signal, we further compared the SERS signals from the graphene-covered and uncovered area. As shown in Figure S6, we found the signals from graphene covered areas displayed relatively higher intensities. Compared with other commonly used gold nanoparticles (60 nm), our substrates usually generated 2 ~ 3 orders of magnitude higher signals (see Figure S7), suggesting the potentiality of our substrate in trace-level organic compounds analysis.

**Figure 3:**
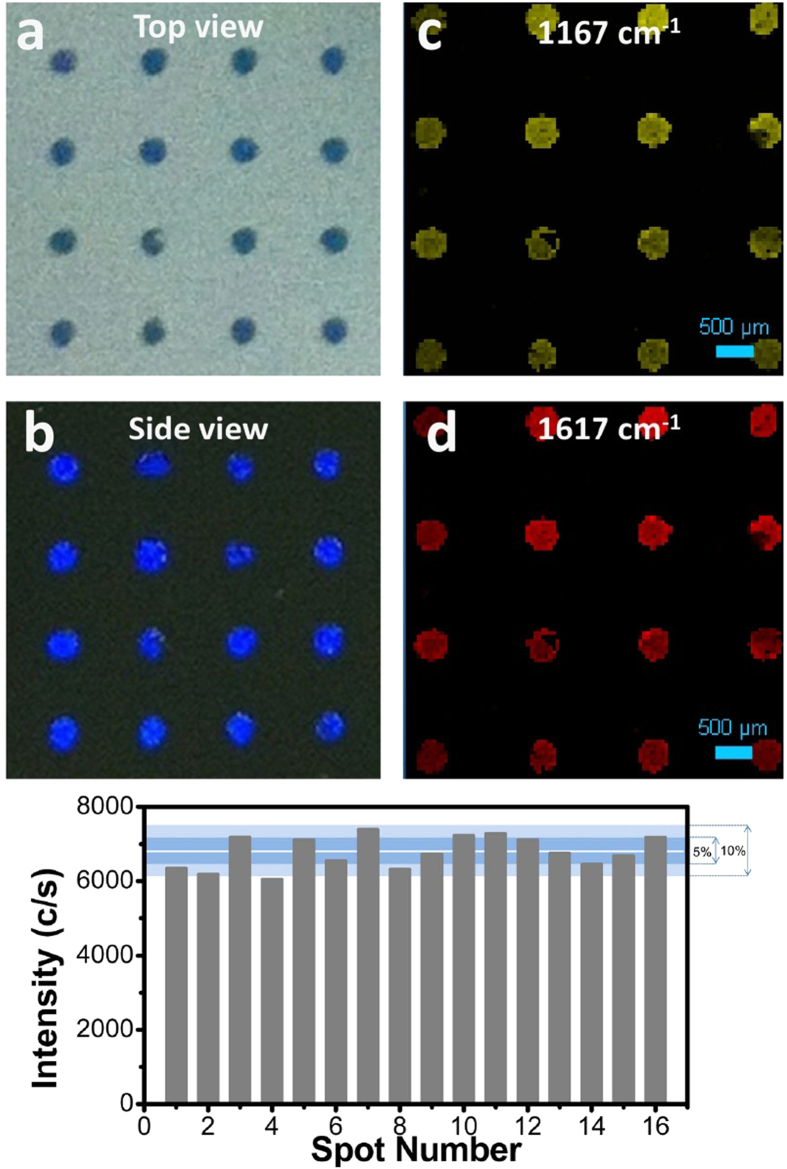
Photographs of an 4 × 4 graphene-shielded SERS substrate array from a different angle (a, b) and SERS intensity mapping collected in an 5 × 5 mm area with a step size of 50 μm (acquisition time for each point is 0.1 s) after the substrate was treated with 10 μM CV and washed thoroughly, which were constructed based on the band intensities at 1167 cm^−1^ (c) and 1617 cm^−1^ (d). (**e**) Average intensity distribution of the 1167 cm^−1^ peak of the 4 × 4 SERS sensing spots (average of 20 randomly selected locations for each spot with 1s acquisition time, using 10× objective). The dark blue and light blue zones represent ±5 and ±5 ~ 10% intensity variation, respectively.

Sensor arrays are practically useful in high-throughput or multi-analyte analysis^28^,^29^,^30^. Interestingly, graphene-shielded SERS sensing spots array could also be prepared by a similar approach. Before silver coating, a facile technique was adopted to generate the SERS sensing array. The excessive nanospheres on Si wafer were removed by an adhesive tape with predrilled patterned holes, resulting in a patterned nanospheres spot array. After silver coating and graphene transferring, the graphene-shielded SERS sensing array was obtained. Figure 3a shows a typical 4 × 4 array containing 16 sensing spots. Each spot was ~500 μm in diameter with an average center-to-center distance ~1.5 mm. The iridescent structural colors from each spot were easily identified (Fig. 3b), suggesting the homogeneous morphology and prominent plasmonic properties of the substrate. But, monolayer graphene on the substrate was hard to distinguish by naked eyes, due to its low color contrast. We collected SERS spectra of the obtained graphene-shielded sensing array after exposure to same concentration of CV. The SERS mapping based on the intensities at 1167 cm^−1^ and 1617 cm^−1^ are shown in Fig. 3c,d, which were perfectly matched the optical images of the array (Fig. 3a,b). Further, to investigate the signal reproducibility of the sensing array, the SERS spectra collected at 20 randomly chosen points (for each spot) are shown in Figure S8. The average Raman intensities of the characteristic 1617 cm^−1^ peak are also quantitatively displayed in Fig. 3e, which shows that 7 of the total 16 sensing spots exhibiting an intensity variation within 5%, while the rest 9 spots are ~10%. The relative standard deviation of the averaged intensity is 6.4%, indicating homogeneous site enhancement distribution and potentialities of the array in quantitative analysis.

**Figure 4:**
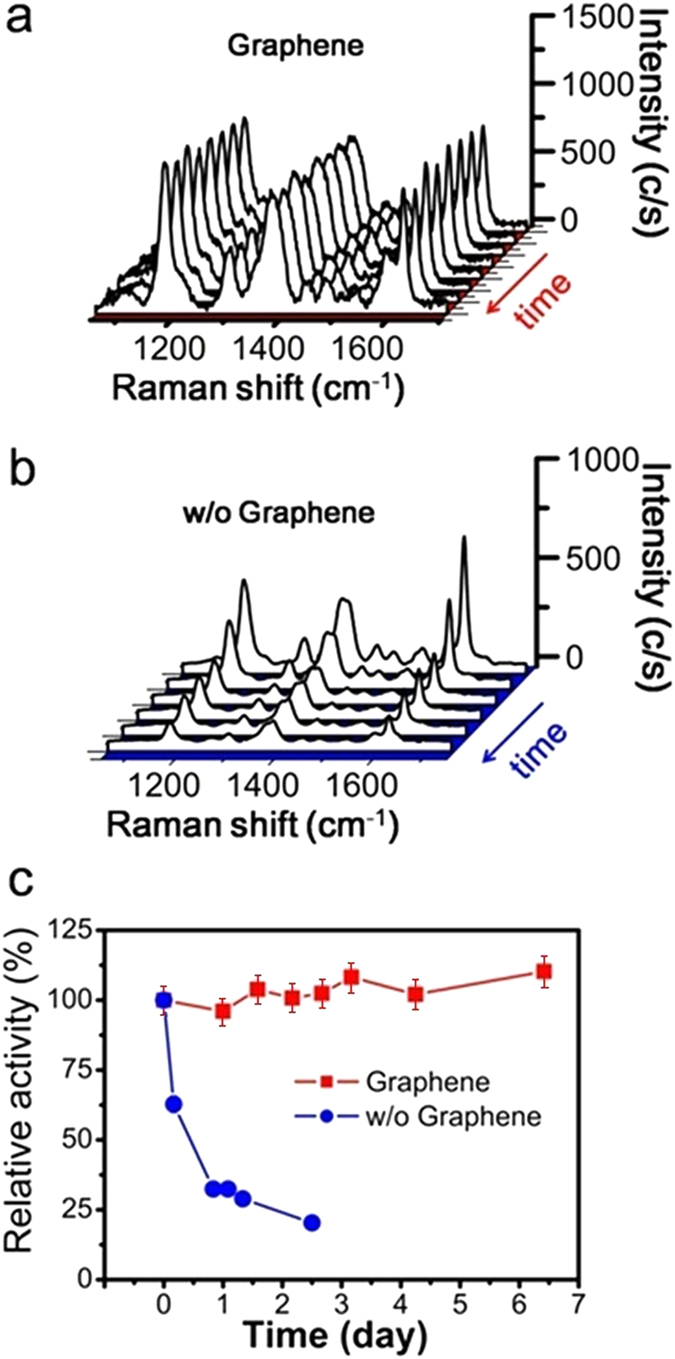
SERS spectra of CV from the SERS substrate with (a) and without (b) graphene protection at different time points. For the substrate without graphene protection, the weakening of SERS intensities were observed with increasing aerobic exposure duration; (**c**) The variation of the SERS intensities at 1167 cm^−1^ cm^−1^ versus the time of aerobic exposure (normalized the SERS intensities before the exposure).

Besides SERS activity and signal reproducibility, the long-term stability is of most concern in practical applications. Despite its effectiveness in generating SERS, the poor stability of Ag nanostructure (against oxidation in particular) undermines its potential in this aspect. To evaluate the stability of our graphene-shielded SERS substrate against oxidation, the substrate was exposed to air at room temperature after soaking in CV solution and dried. The Raman spectra were collected at different time intervals (Fig. 4a). For comparison, the SERS substrate without graphene coating were tested under same conditions (Fig. 4b). For the uncovered substrate, we noticed the degradation of the signal began after very short exposure. Quantitatively, as indicated in Fig. 4c, the signal dropped to ~60% of its initial value after 2 h and dropped to ~30% after 18 h, indicating severe oxidation occurred on uncovered substrate. In contrast, the CV signal from the graphene-shielded SERS substrate maintained their intensities and fine features. It did not suffer any obvious degradation even after 7 days of exposure. The variation of the intensities were less than 10% in this period. It has been reported that graphene- passivated surface can suppress the oxidation and reserve the plasmonic activity of the metallic nanostructures^23^,^24^,^36^, since the seamless graphene is impenetrable to most of the gases, including O_2_. An extreme case is shown in Figure S9, in which displays a graphene-shielded SERS substrate stored in ambient conditions for 8 months. The heavy oxidation caused the uncovered silver film turning dark brown, while in the graphene-covered area, it still shined brightly. This may serve as a vivid example of the excellent anti-oxidation ability of the graphene-coating.

**Figure 5:**
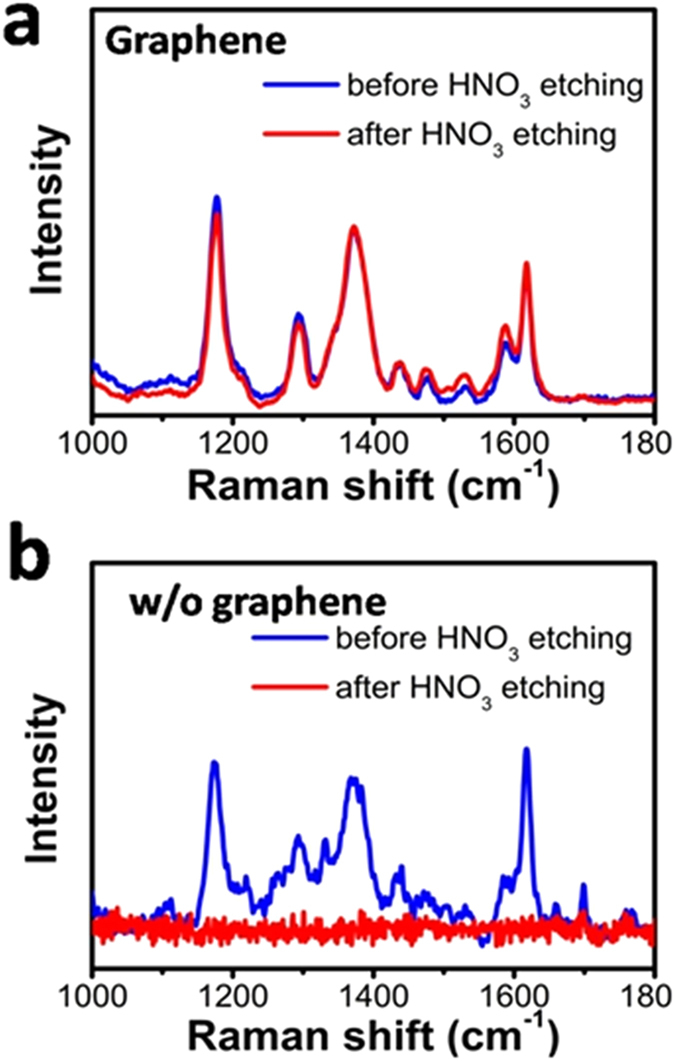
(**a**) Comparison of CV spectra from graphene-shielded SERS substrate before and after treated by 6.5% HNO_3_ for 1 min; (**b**) As a control test, the SERS substrate without graphene protection was measured under same conditions. The CV signal completely vanished after the etching by HNO_3_.

Direct exposure of the silver-based SERS substrate to non-ideal environment may also cause degradation of SERS activity, due to the chemically instability of Ag. But, we found that the graphene-shield SERS substrate could be stable in those environments. Figure 5a shows the CV spectra on a graphene-shielded substrate after etching by concentrated HNO_3_ (6.5%) for 1 min, which did not show obvious decay. In contrast, the signal from the uncovered substrate completely vanished after etching (Fig. 5b). The above experiment clearly indicated that graphene can protect the SERS substrate against HNO_3_ etching. This feature may expand the use of SERS substrate in some non-ideal conditions, especially when corrosive reagents are involved.

**Figure 6:**
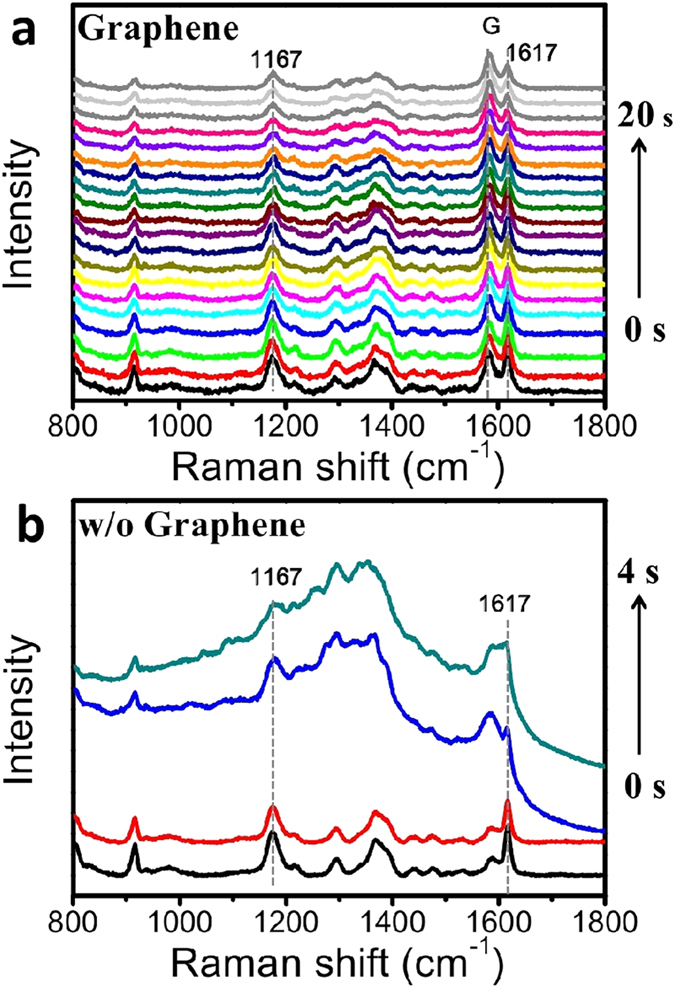
Comparison of thermal stability for the substrates with (a) and without (b) graphene protection under continuous laser excitation. An obvious photocarbonization effect was observed in the substrate without graphene protection after 3 s of excitation. Laser power density is 2 × 10^8^ mW/cm^2^. Each spectrum has an acquisition time of 1 s.

As an adverse side-effect of the enhanced LSPR adsorption, photocarbonization and photobleaching are inherent and frequently encountered problems in the SERS measurements^37^. In this work, we observed the graphene coating could alleviate these photo-induced damage to both the substrate and the analyte. As demonstrated by following test, we repeatedly measured the SERS spectra at a fixed point on the substrate under continuous laser excitation. Figure 6a shows the spectra during a period of 20 s illumination (laser intensity is about 1.5 × 10^8^ mW/cm^2^). The CV signal on the graphene-shielded substrates remained stable. Under same conditions, for the uncovered substrate, an obvious photocarbonization occured only after 3 s of illmunination (Fig. 6b). Even illuminated with lower laser intensities (7.5 × 10^7^, 3.8 × 10^7^ mW/cm^2^), the photocarbonization still happened after 5 s and 10 s, respectively (data not shown). This may attribute to the extremely high thermal conductivity^38^ of the graphene coating, due to its long phonon mean free paths. Thus, this feature may also help to improve the reproducibility of SERS measurement by reducing the signal fluctuation caused by photo-induced damage.

**Figure 7:**
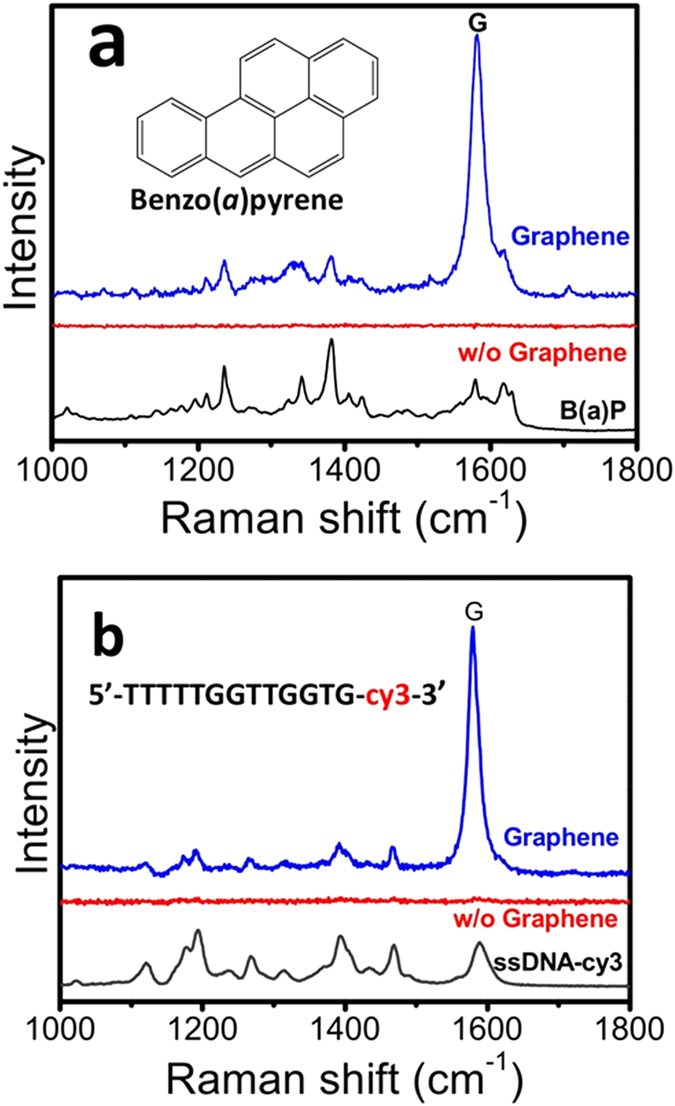
(**a**) SERS spectra after the substrates were immersed in (**a**) 10 ppm B(a)P and (**b**) 1 μM Cy3-labeled ssDNA in 0.1 M NaCl solution for 1 h and thoroughly washed by 0.1 M NaCl. The SERS spectra of B(a)p and Cy3 labeled ssDNA are also presented in the same figures (black).

Pervious work indicates SERS are effective in detecting molecules strongly adsorbed on the SERS substrate (pyridine, thiols and heterocyclic molecules, *etc.*), but for molecules with little affinity, SERS becomes rather ineffective^39^. Since graphene shows high affinity to the aromatic molecules and biomolecules that are weakly adsorbed on conventional metallic SERS substrate^40^, our graphene-shielded SERS substrate may help to solve the problem. We chose benzo(a)pyrene (B(a)P) and ssDNA (dye labeled) as models to study the enrichment effect of the substrate. B(a)P is a polycyclic aromatic hydrocarbon, a common byproduct of incomplete combustion or burning of organic components, which is classified as mutagen and potential carcinogen. Due to its high hydrophobicity and low affinity to the metallic surface, it is difficult to detect B(a)P *via* conventional SERS substrate^41^,^42^. On the other hand, the specific recognition of DNA has been widely used in biosensing *etc.*, and thus detection of DNA is also of great importance. Due to the inherently weak Raman signal of DNA, we used a dye labeled ssDNA instead. As shown in Fig. 7, the B(a)P or DNA exposed onto the graphene-coated substrate exhibited a stronger Raman signal than that of uncovered ones, indicating the existence of certain enrichment effect (perharps due to the π - π stacking interactions). It should be noted this effect is case-sensitive. But, a lower detection limit for certain analytes can be expected. More importantly, this effect may expand the applicability of SERS technique to molecules with little affinity to bare SERS substrates.

## Discussion

SERS applications are highly dependent upon the SERS-active substrate. The basic goal of designing SERS substrates is to maximize enhancement factor while maintaining good signal reproducibility. Besides, the stability of the SERS substrates is of most concern. One common problem is the degradation of SERS substrates caused by oxidation or corrosion, which is hard to solve by conventional coating methods^10^,^12^,^13^. In this study, we present a graphene-shielded periodic metallic nanostructure as the SERS substrate. By passivating the surface with a monolayer graphene, the substrate exhibited a long-term stability against oxidation (Fig. 4). We also noticed that the graphene could protect the substrate in highly corrosive environment (Fig. 5). It would be reasonable to assume that such protection effect should be valid for other reactive compounds in air or liquid, since CVD-grown graphene is impenetrable to most gas molecules and liquids^16^,^17^, An proof can be found in a recently study that sulfidation of Ag caused by H_2_S can also be prevent by the graphene^36^. All these results suggested the significant role of graphene in improving the stability of the SERS substrate, either in storage or usage.

Besides acting as chemically inert coating, graphene also endows many novel features to the substrate. For one thing, the graphene passivated substrate tends to provide a smoother surface with more well-defined molecule-substrate interaction, which may also help to improve the reproducibility of the SERS measurement^22^. Secondly, the extremely high thermal conductivity of graphene allows swiftly dispersing the heat generated by laser excitation. Thus, the graphene can reduce the photo-induced damage to the substrate or the analytes (Fig. 6) and also provides a cleaner baseline^4^,^37^,^43^. This feature also implies a higher illumination power could be used on graphene-shielded substrates. Thus, an improvement in the signal-to-noise ratio or detection sensitivity could be expected. Thirdly, due to the π - π stacking interactions, graphene show high affinity to many aromatic molecules and biomolecules that are weakly absorbed to conventional SERS substrate, suggesting those molecules could be effectively detected by graphene-shielded substrate. Finally, in principle, the substrate-analyte isolation strategy offers protection not only to the substrate, but also to the analyte. For instance, Ag^+^ ion is proven to be toxic to many biomolecules^44^. Since graphene-shielded substrate was stable in corrosive environment (shown in Fig. 5), it is reasonable to assume that the graphene could restrain the release of Ag^+^ ions of the substrate to environment and thus protect the biomolecules. Especially, graphene exhibits excellent biological compatibility. These features make graphene-shielded substrate a promising platform for monitoring the biological process.

The lack of reliable and cost-effective fabrication technique for high performance substrate remains a bottleneck for SERS applications. In this work, we utilized a metal-coated nanospheres arrays as SERS substrates. Compared to with lithography-based methods^5^,^6^,^7^,^8^, it allows to achieve considerable high and homogenous SERS enhancements at affordable cost (shown in Figs 2 and 3). The other advantage is its tunability of SERS activity. By adjusting the diameter of nanosphere beads, the LSPR of the substrate can be further optimized for specific excitation wavelength (Figure S10, S11)^45^. Moreover, since the fabrication of larger-area ordered nanospheres array is not difficult and industrial scale CVD-grown graphene are widely available, it is possible for our approach to be developed into a scalable method in future.

We also managed to prepare graphene shielded SERS sensing array via a similar approach, as indicated by Figure S2. Sensor arrays are a practically useful in high-throughput or multi-analyte analysis^28^,^29^,^30^. One of the advantages of our SERS sensing spots array is the low-sample volume (<0.5 μL) requirement for each measurement. The hydrophobic surface of the graphene prevented the sample droplet from spreading over the entire area, thus allowing the analyte concentrating on each spot. Some on-site screening tests could be developed based on our SERS sensing array, such as for drug abuse, explosive, environmental hazards, *etc.* On other hand, since graphene can be easily functionalized with bioreceptor units^18^,^46^, the array could also serves as a multi-analyte biosensing platform. All these aspects are certainly worth further studying.

Besides SERS, other optical properties of the graphene-shielded substrates also caught our attention, such as the LSPR. Intense confined electromagnetic fields induced by the LSPR are capable of detecting small changes in the dielectric environment around the nanostructures^47^. Combined with the high stability endowed by graphene-passivated nanostructure, it would sound attractive in LSPR-based biosensing.

Altogether, we concluded that a graphene passivation layer on the SERS substrate has dramatic effect on its properties and morphology of the substrate. The unique structural features and properties of graphene exactly compensate some drawbacks of the conventional metallic SERS substrates. It provided a chemical inert surface for the SERS substrate to fight against oxidation and chemical etching, and gave many new features. Furthermore, the proposed graphene-shielded SERS sensing array could be developed into high-throughput analysis or multi-analyte biosening. All these results indicated that this new SERS substrate holds great promise both in fundamental studies of the SERS effect and many practical fields.

## Methods

### Materials

CVD-grown signal layer graphene on copper foil (5 × 10 cm) was purchased from ACS Material, LLC. Monodisperse Polystyrene (PS) microspheres with diameter 400, 500, 600, 700, 1000 nm were obtained from BaseLine Co. (2.5% w/v, CV less than 5%). Poly(methyl methacrylate) (PMMA) was brought from GERMAN TECH (average MW 950,000, dissolved in ethyl lactate). Poly (ethylene oxide) (PEO) with MW 10 k, sodium dodecyl sulfate (SDS) and ammonium hydroxide were purchased from Sigma-Aldrich. Other chemicals, propanol, CuSO_4_, HCl, H_2_SO_4_, H_2_O_2_, were obtained from Sinopharm Chemical Reagent Company. The above reagents were uses without further purification and Milli-Q water (18 MΩ·cm^−1^) was used to prepare all anqueous solutions. All silica wafers or glass slides were cleaned in a freshly prepared piranha solution (3:1, 98% H_2_SO_4_/30% H_2_O_2_) for 10 min and in 1:5:1 NH_3_H_2_O/H_2_O/H_2_O_2_ solution for 1 h. The substrates were then rinsed by excessive H_2_O and stored in Mili-Q H_2_O until use.

### Fabrication of Metal-Coated Nanospheres Arrays SERS Substrate

Large-area two-dimensional (2D) crystalline colloidal arrays was fabricated as previous report^31^,^48^. Silica and PS nanospheres were firstly separated by centrifugation, washed by excessive H_2_O several times and were redispersed in H_2_O again. Then, the nanopheres dispersion (10 wt%) was mixed with isopropanol (2:1, v/v) and with a trace amount of PEO (2 mg mL^−1^). The nanopheres dispersion was vortexed for at least 3 min before injected into water-air interface by a syringe pump (Cole-Parmer) and formatting of two-dimensional ordered nanopheres array at the interface. To transfer the 2D nanopheres array onto a silica wafer or glass slide, we lifted up the pre-placed the substrate or drained the water in the container, The residual solvent on the substrate was then allowed to evaporate in ambient conditions, during which the nanopheres formed a close-packed array. Before metal coating, the prepared monolayer of nanospheres on the substrate was annealed in an oven at 80 °C for 1 h. A 200 nm thick silver film was deposited at a rate of 0.83 Å·s^−1^ under vacuum (3 × 10^−2^ mbar) by a sputter coater (Quorum Q150R ES). The substrates were spun at 100 rpm during deposition. The metal film thickness was measured by a step profiler (Veeco Dekak 150). To generate the SERS sensing spots array, the excessive PS was removed by an adhesive tape (Scotch, 3M) with predrilled patterned holes, which was subsequently coated with the silver film.

### Graphene Transfer Procedure

CVD-grown graphene was transferred on the metal-coated nanospheres arrays by following procedure. The graphene film on Cu foil was firstly spin coated with PMMA (500 rpm for 5 s and 5000 rpm for 1 min) and then cured at 105 ^o^C for 90 s. Thus, the Cu foil was etched away in an aqueous CuSO_4_/HCl solution for 4 h. The resulting PMMA/graphene film was washed with Mili-Q water several times and was then transferred onto the desired SERS substrate. After the substrate was completely dried, the substrate was further dried in a vacuum oven at 50 °C for 30 min. Then, the PMMA layer was removed by chloroform and acetone. Then the substrate was rinsed by sopropanol, water and dried in a gentle N_2_ flow.

## Additional Information

**How to cite this article**: Liu, X. *et al.* Compact Shielding of Graphene Monolayer Leads to Extraordinary SERS-Active Substrate with Large-Area Uniformity and Long-Term Stability. *Sci. Rep.* **5**, 17167; doi: 10.1038/srep17167 (2015).

## Supplementary Material

Supporting Information
