# Supplementary material for: Compact Shielding of Graphene Monolayer Leads to Extraordinary SERS-Active Substrate with Large-Area Uniformity and Long-Term Stability
Source: Sci Rep. 2015 Nov 30;5:17167. doi: 10.1038/srep17167 (PMC4663485; doi:10.1038/srep17167)
Supplement: Supporting Information [file srep17167-s1.doc]

Compact Shielding of Graphene Monolayer Leads to Extraordinary SERS-Active Substrate with Large-Area Uniformity and Long-Term Stability

Xiangjiang Liu,^†^ Longhua Tang,*^,‡^ Yichen Wu,^‡^ Tianren Fan,^‡^ Yang Xu,^§^ and Yibin Ying*^,†^

^†^ College of Biosystems Engineering and Food Science, Zhejiang University, Hangzhou 310058, China.

^‡^ State Key Laboratory of Modem Optical Instrumentation, Department of Optical Engineering, Zhejiang University, Hangzhou 310027, China

^§^ Institute of Microelectronics and Optolectronics, Zhejiang University, Hangzhou 310027, China

* Corresponding author: Email: [lhtang@zju.edu.cn](mailto:lhtang@zju.edu.cn); [ibeying@zju.edu.cn](mailto:ibeying@zju.edu.cn)

KEYWORDS. Surface-enhanced Raman scattering, graphene，array, silver, stability, reproducibility


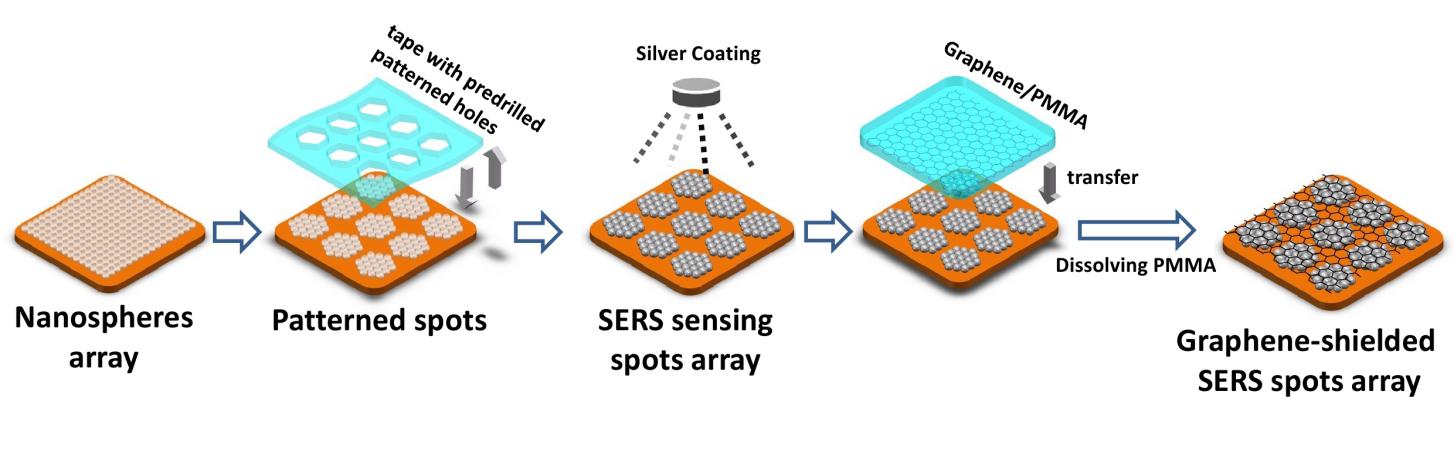


**Figure S1**. Schematic route for sample preparation with graphene-shielded SERS substrate array.


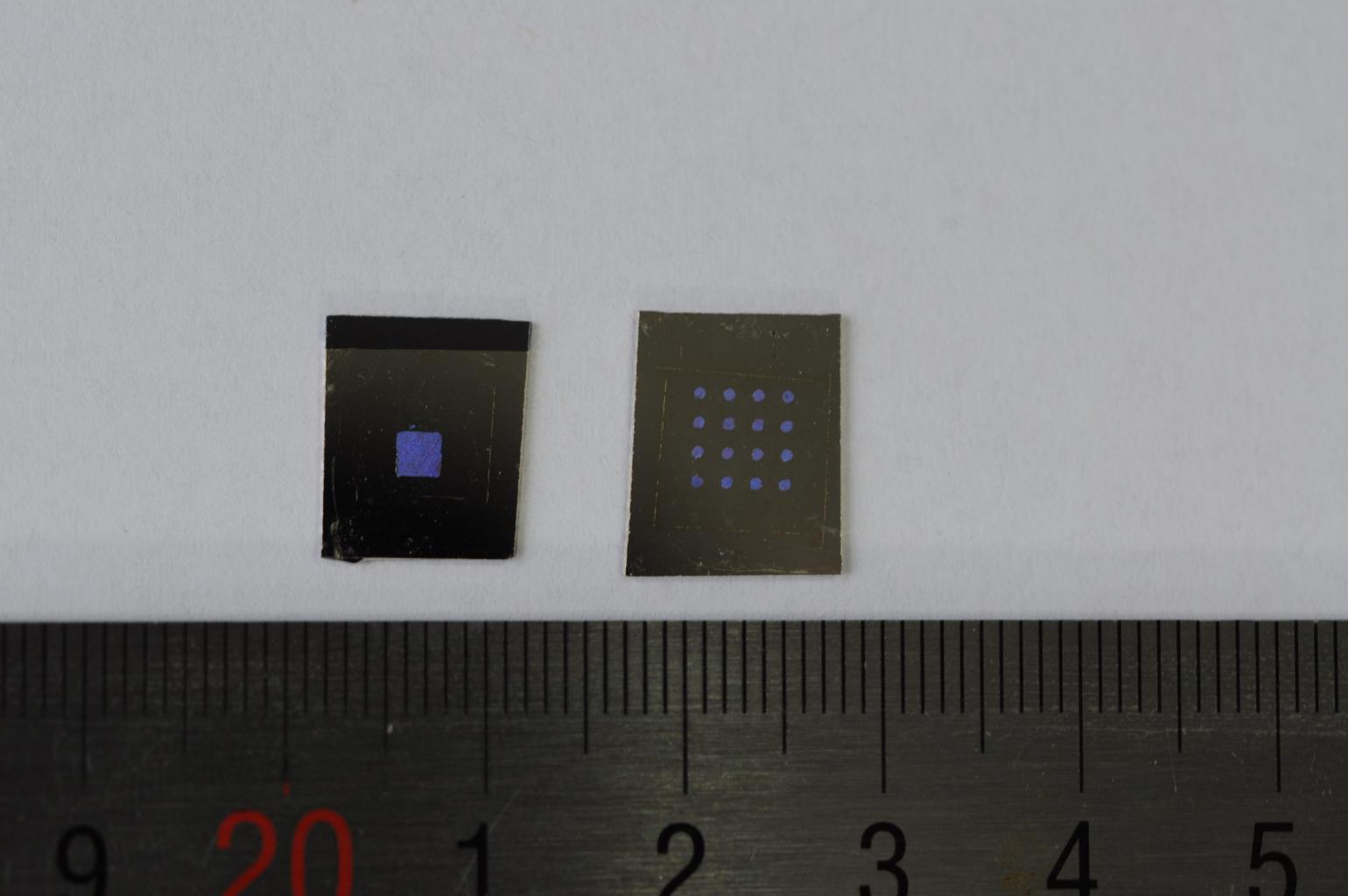


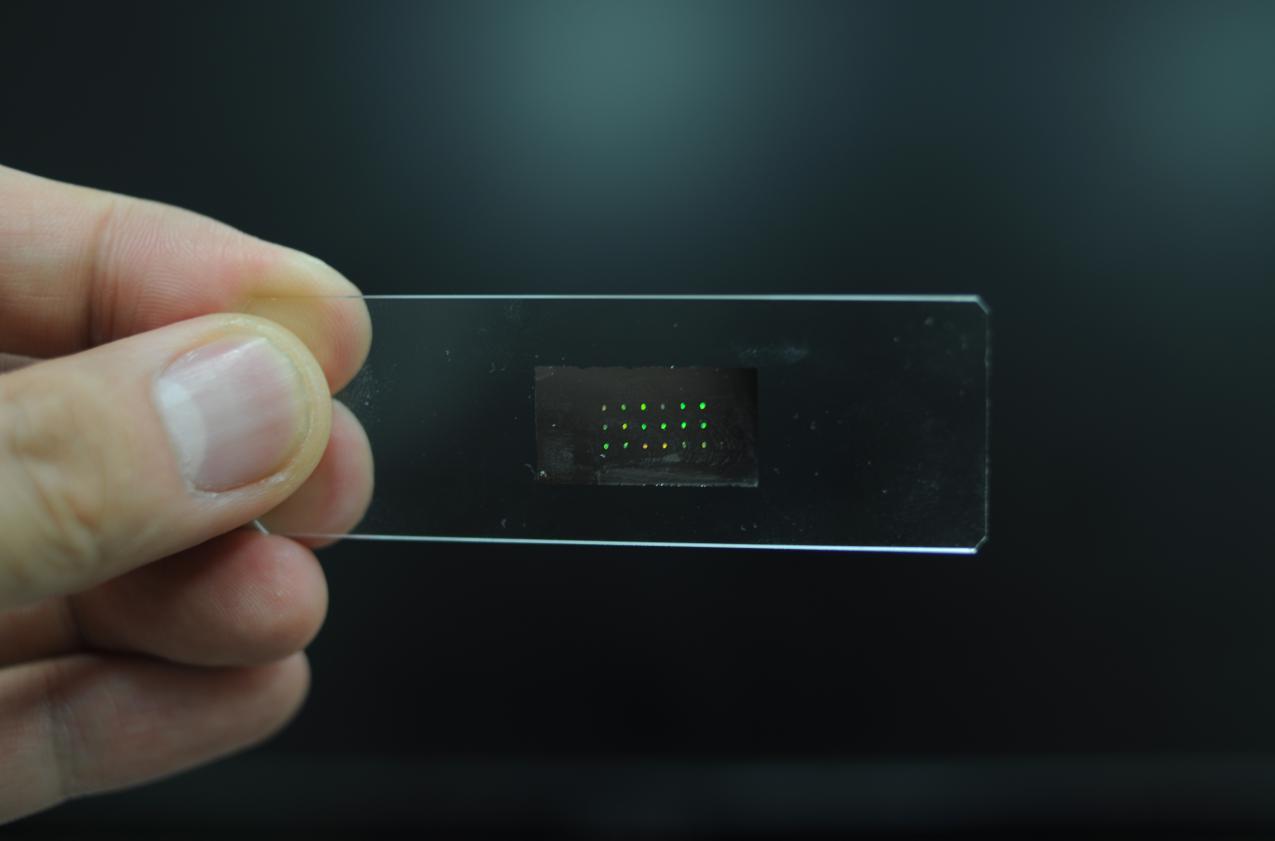


**Figure S2.** Typical images of graphene-shielded SERS substrates.


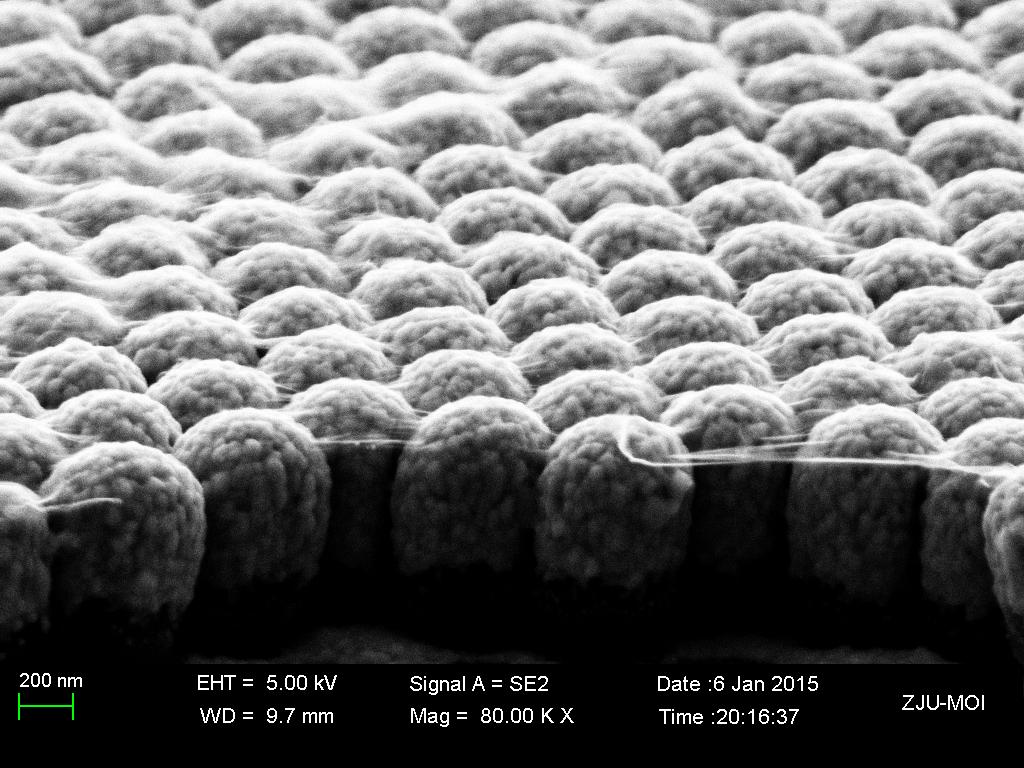


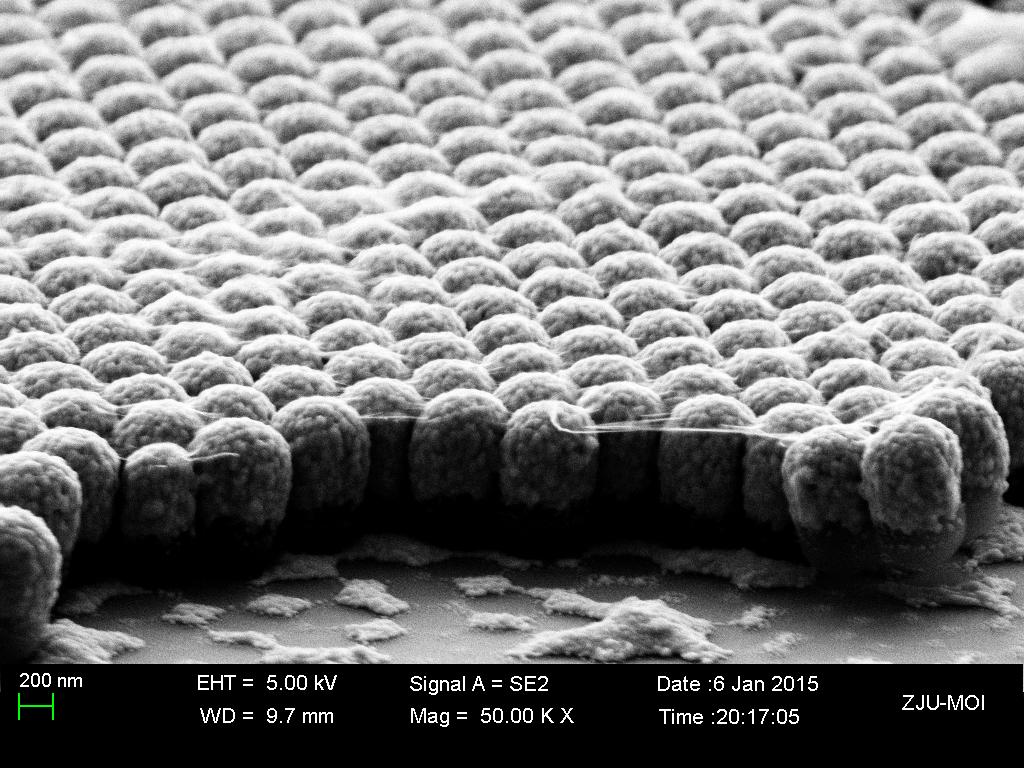


**Figure S3.** SEM images of the cross section of graphene-shielded SERS substrate array


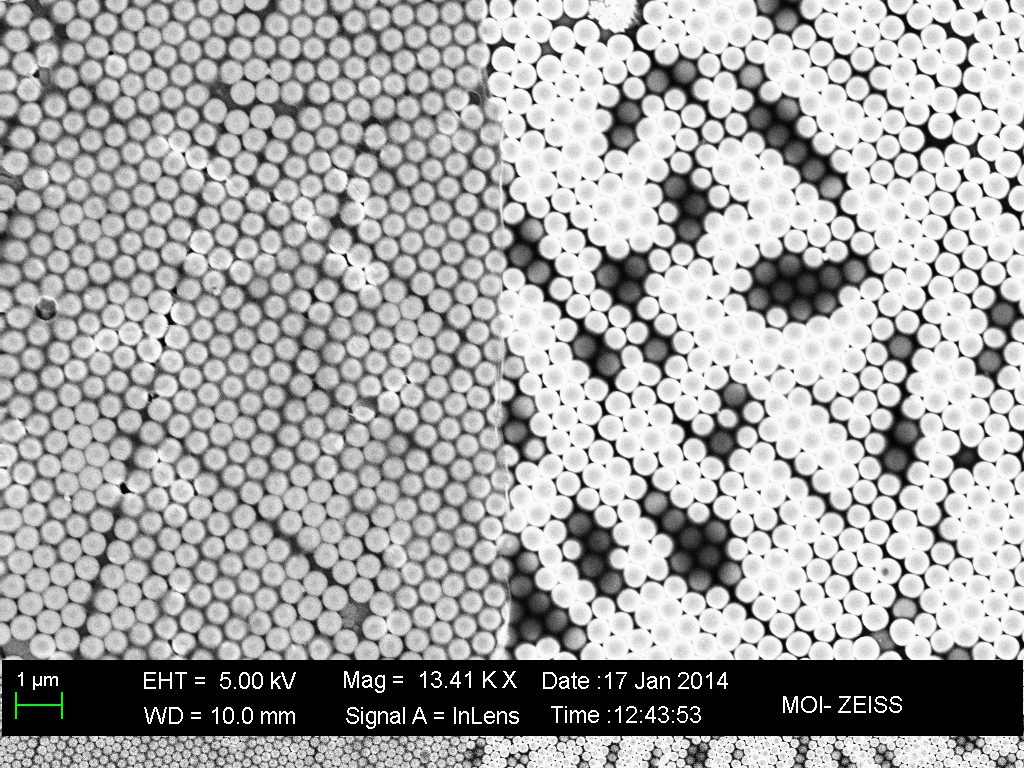


**Figure S4.** Top view of the graphene-shielded SERS substrate.


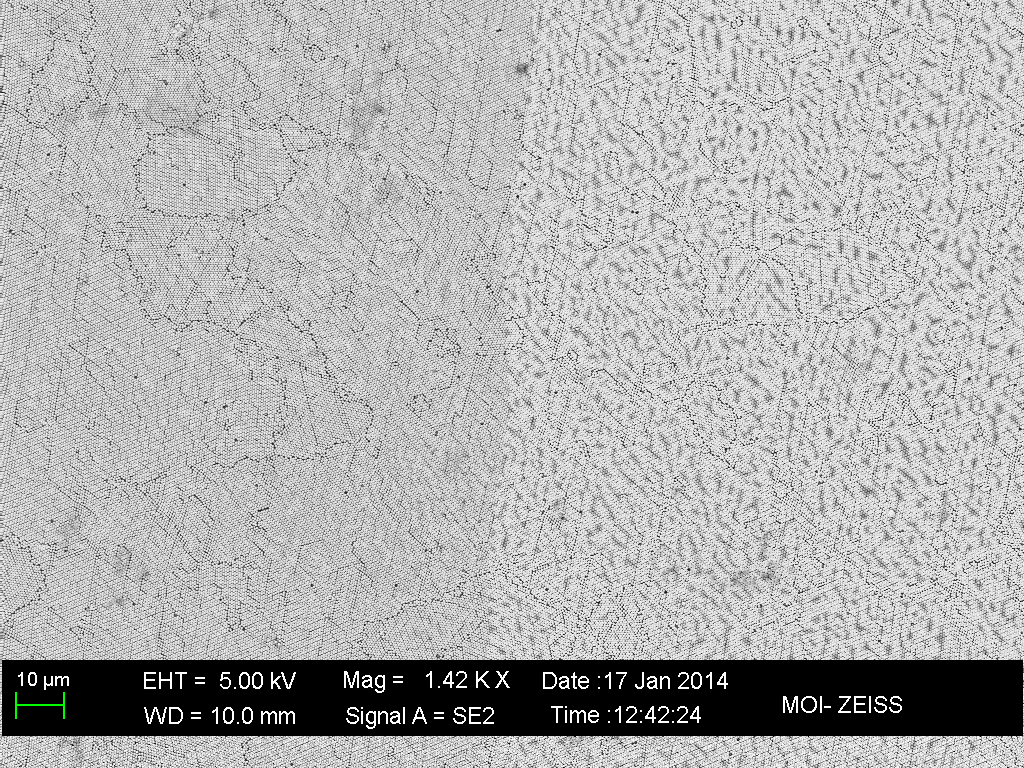


**Figure S5.** SEM images of the large-scale graphene-shielded SERS substrate array

**
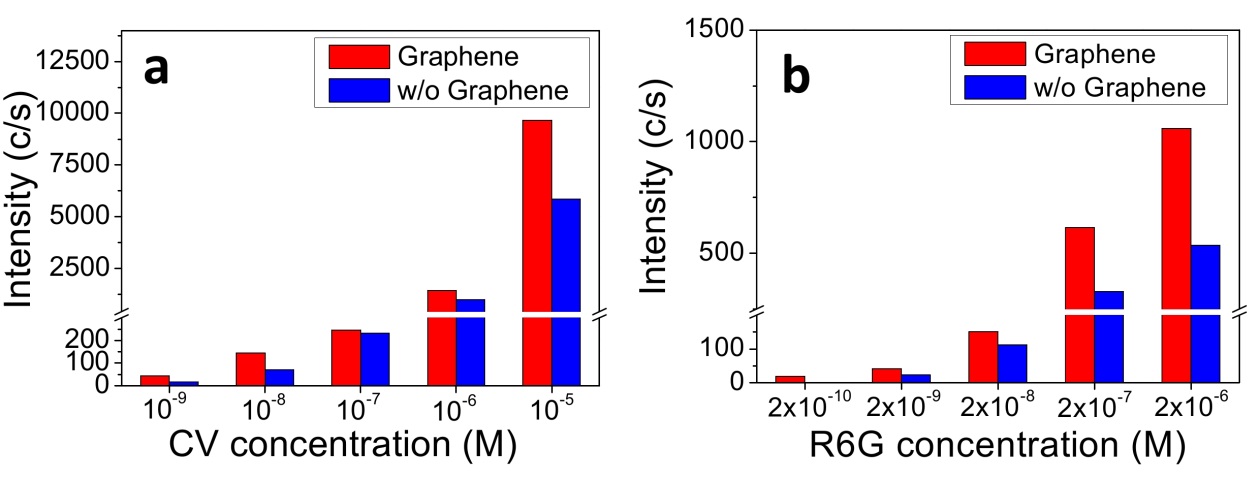
**

**Figure S6** Comparison of SERS intensities recorded on the graphene-covered and uncovered area. (50× objective, average of 16 spectra with 1s acquisition time).

**Figure S7** Comparison of SERS intensities (1617 cm^-1)^ of CV (top) and R6G (down) recorded on the graphene-shield substrates (50× objective, average of 16 spectra with 1s acquisition time) and 60 nm gold nanoparticles (50× objective, average of 5 spectra with 10s acquisition time).


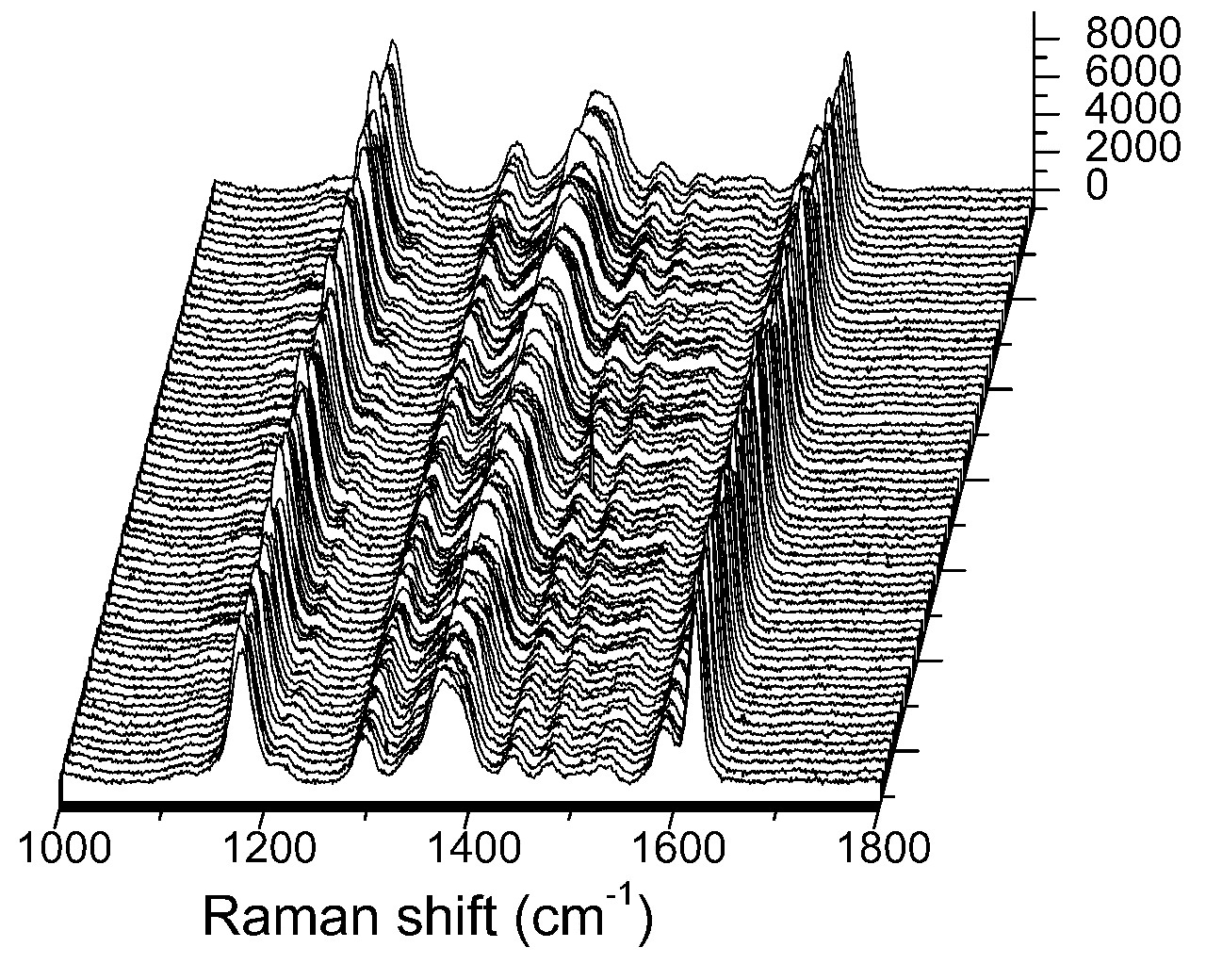


**Figure S8** Raman spectra on the SERS substrate containing 16 sensing spots.


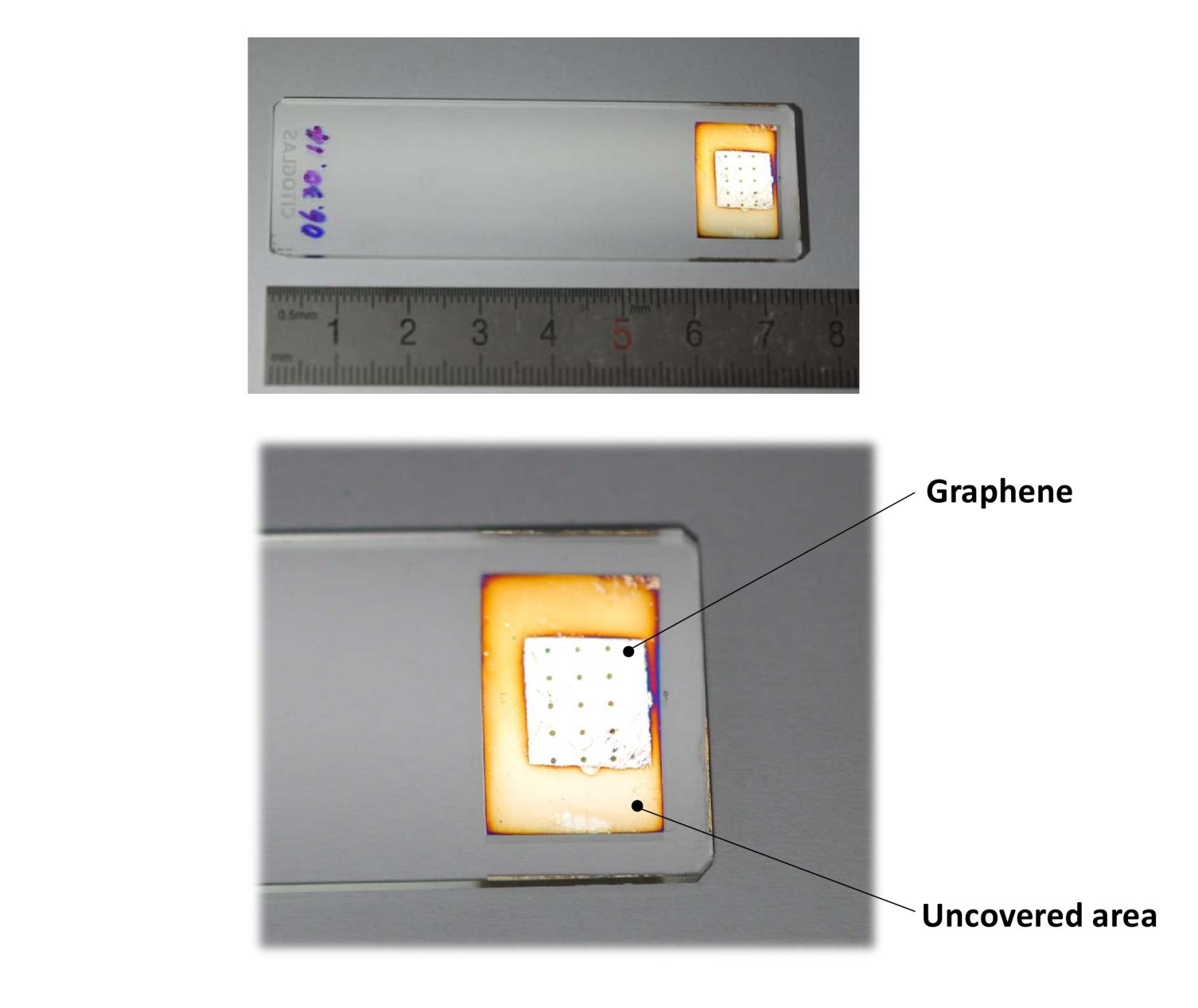


**Figure S9** Image of a graphene-shielded SERS substrate after 8 months storage in ambient air since its preparation. The brown color in the uncovered area. indicates severe oxidation occurred. In contrast, in the graphene-covered area, it is still shining brightly.

**Figure S10.** Adsorption spectra of the graphene-shielded SERS substrates with different nanosphere diameter.

**Figure S11.** The plot of the SERS intensities at 1167 cm^-1^ of CV versus the nanosphere diameter. The laser of 632 nm was used.
